# Miscellanies

**Published:** 1821-12-01

**Authors:** 


### MISCELLANIES.

We believe it is the intention of the members of the MEDICO-CHIRURGICAL SOCIETY of London, to dine together in the month of January, 1822. The number, respectability, and influence of its members will, in all probability, cause this meeting to present the greatest assemblage of rank, talent, and learning in the profession, that was ever witnessed at any one time or place. If, on such an occasion, the "feast of reason and the flow of soul" do not predominate, it will be wonderous strange. We hope our worthy president, Dr. Cooke will be able to take the chair. No man is better qualified to fill it, by a union of the erudition of the scholar, the dignity of the philosopher, and the refined manners of the gentleman.

*Medico-Chirurgical Society of Edinburgh.*—It is with pleasure that we announce the formation of a Medico-Chirurgical Society in Edinburgh. The society is formed upon the model of the Medico-Chirurgical Society of London; and has in view precisely similar objects. Most of the medical Professors in the university, and many of the most respectable Practitioners in the city, have co-operated in its formation. Dr. Duncan, Sen. has been elected its first president. Its sittings commence in the approaching winter session.

In addition to ordinary and honorary members, provision is made for the admission of corresponding members; and it is hoped that many, in almost every part of the world, and such especially as retain a grateful recollection of the advantages they derived from their alma mater, will not be backward in supplying interesting communications.

Communications may be transmitted to the president of the society, or to either of the secretaries, according to the following addresses:

Dr. W. P. ALISON, 44, Heriot Row, Edinburgh.

Dr. ROBT. HAMILTON, 3, Northumberland Street, Edinburgh.

A physician, a graduate of the University of Edinburgh, and Member of the Royal College of Surgeons in London, residing in a populous country town, has a vacancy in his family for a young gentleman who may have finished his apprenticeship, and intends taking his degree in one of the Universities. He will have ample opportunity of practice in a Public Institution, and recourse to a good medical library. It will be required of him to devote a certain portion of each day to his professional studies, on which he will have to undergo regular examinations. He will be treated as one of the family. Terms, 150 guineas per annum.

For further particulars apply to Dr. Johnson, No. 58, Spring Gardens.

### *Additional Subscribers since last Quarter.*

